# Cochlear Ossification in a Patient with Cogan's Syndrome Undergoing Bilateral Cochlear Implantation

**DOI:** 10.1155/2018/7395460

**Published:** 2018-11-11

**Authors:** Adam M. Cassis

**Affiliations:** West Virginia University, Department of Otolaryngology, 1 Medical Center Drive, P.O. Box 9100, Morgantown, WV 26505-9600, USA

## Abstract

We present the case of a young female patient diagnosed with Cogan's syndrome after the rapid onset of profond hearing and vestibular loss with concomitant eye symptoms. After appropriate medical treatment, her hearing did not respond and she underwent bilateral simultaneous cochlear implantation with findings of extensive cochlear ossification in both ears. The case and outcome are described in the body of the paper.

## 1. Introduction

Cogan's syndrome is a rare condition consisting of recurrent visual and auditory-vestibular deficits, causing patients to complain of visual loss, eye pain, hearing loss, tinnitus, and vertigo. The hearing loss can be progressive and lead to a severe to profound hearing loss in 50–85% of cases [1–4]. This hearing loss may be associated with cochlear ossification, which may prove challenging if cochlear implantation is required for auditory rehabilitation. The following case describes a patient with sudden onset hearing loss and vertigo with associated eye symptoms who ultimately required bilateral cochlear implantation with findings of severe cochlear ossification in both ears.

## 2. Case Report

A 24-year-old female presented to the otology clinic with a seven-week history of sudden onset right sided hearing loss and vertigo. One week later, she suffered left sided hearing loss. One month after the onset of her symptoms, she was evaluated by a community otolaryngologist who suspected Cogan's syndrome (CS) due to concurrent blurry vision, photophobia, eye pain, and excessive watering. She was treated with high pose prednisone and referred to our center for evaluation by otolaryngology, ophthalmology, and rheumatology. Her past medical history was significant for asthma.

Ophthalmology observed subepithelial corneal infiltrates, but they were not felt to be consistent with classic interstitial keratitis. She was prescribed steroid eye drops, and her vision and pain improved dramatically. Rheumatology felt she had atypical CS and started treatment with methotrexate.

**Figure 1:**
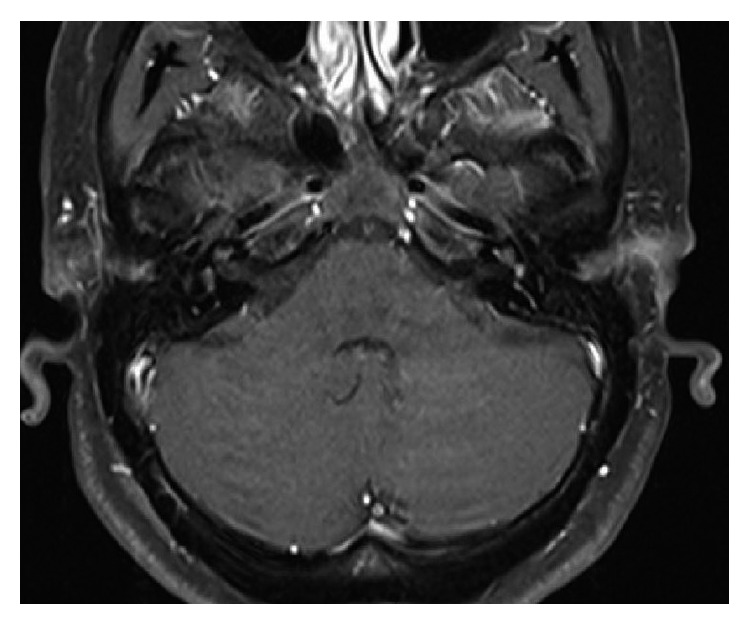
MRI T1 axial postcontrast imaging demonstrating bilateral enhancement of the cochlea, vestibule, and semicircular canals.

On presentation to clinic, her vertigo and imbalance had mostly resolved; however, her hearing did not improve. Initial audiogram and repeat audiogram after prednisone showed profound bilateral hearing loss with 0% speech discrimination bilaterally. MRI scan revealed enhancement of the otic capsule bilaterally (Figure 1). At the time of her scan, there was no loss of fluid signal from the cochlea on FIESTA sequencing as might be expected if cochlear fibrosis and/or ossification were to be present. Autoimmune serology labs were normal. Given the lack of response to medication and duration of her sudden onset hearing loss of 7 weeks, we felt she would benefit from simultaneous cochlear implantation, and the patient agreed to the procedure.

At surgery, patient was found to have significant ossification of the scala tympani on both ears. Fortunately, a full electrode insertion was completed on each side after a significant basal turn drill-out was performed (Advanced Bionics HiRes Ultra device with mid-scala electrode, Valencia, CA). Five weeks after surgery, she was appropriately healed, and her devices were activated. During activation, elevated impedances were found on the right at electrodes 3, 12, 13, and 15, while the left side showed normal impedances. The increased impedances slowly decreased over time and are currently within the normal range, although elevated compared to the remainder of the electrodes. Currently, the patient is using cyclosporine drops in both eyes, both of which have good vision and are without pain. She continues to follow with rheumatology, who has prescribed a maintenance dose of methotrexate at 20 mg weekly. Five months after activation, she attained a word recognition score of 76%. She continues to show improvement in her implant performance without any signs of decrement.

## 3. Discussion

A rare entity, CS causes vision loss, hearing loss, and dizziness. Although the cause is unknown, it is thought to be due to an autoimmune process that attacks these tissues. This usually occurs in patient in the third to fourth decade of life. Physical exam findings of nonsyphilitic interstitial keratitis on slit-lamp examination of the eye, abnormal lab findings (increased white blood cell count, erythrocyte sedimentation rate, and C-reactive protein), and documented hearing loss on audiometry help to make the diagnosis. Treatment usually consists of oral corticosteroids, although if a therapeutic response is not achieved, stronger immunosuppressive drugs may be necessary.

A recent review found that 98% of patients with CS had hearing and/or vestibular symptoms. Of that group, bilateral hearing loss was present in 41% and deafness in 31%. For those patients who suffer unaidable hearing loss, cochlear implantation is necessary to restore sound. This disease process, however, can present challenges to the surgeon. A recent case series of cochlear implantation in CS revealed that half of these patients had ossification of the scala tympani during surgery (6 of 12 patients). Despite these findings, their population of CS implant recipients obtained an average word recognition score of 91% at one year of device use [5].

Since cochlear abnormalities are frequent with CS, preoperative evaluation and planning are crucial to successful implantation surgery. This case demonstrated the severity of cochlear ossification that can occur in CS. This information is useful not only for surgical approach and device selection, but for preoperative counseling with the patient as well. Although the reported follow-up is of short duration (5 months) and there is potential for fluctuation in implant performance over time, she has consistently improved with her implants over time and is yet to observe any decreased performance at the time of writing this manuscript.
